# Obtaining spontaneously beating cardiomyocyte-like cells from adipose-derived stromal vascular fractions cultured on enzyme-crosslinked gelatin hydrogels

**DOI:** 10.1038/srep41781

**Published:** 2017-02-03

**Authors:** Gang Yang, Zhenghua Xiao, Xiaomei Ren, Haiyan Long, Kunlong Ma, Hong Qian, Yingqiang Guo

**Affiliations:** 1 Department of Medical Information and Engineering, School of Electrical Engineering and Information, Sichuan University, Chengdu 610065, China; 2 Department of Cardiovascular Surgery, West China Hospital, Sichuan University, Chengdu 610041, China; 3 Center of Engineering-Training, Chengdu Aeronautic Polytechnic, Chengdu 610100, China; 4 Department of Orthopaedics, Yongchuan Hospital, Chongqing Medical University, Chongqing 402160, China

## Abstract

Heart failure often develops after acute myocardial infarction because the injured myocardial tissue fails to recover or regenerate. Stem cell transplantation using adult cell sources, such as adipose-derived stromal vascular fraction (SVF), draws extensive attention. In this study, SVF cells were isolated from rat adipose tissue and cultivated on enzyme-crosslinked gelatin hydrogels. Morphological features of cell development and spontaneous beating behavior from these cells were observed and recorded. Cardiac phenotypes were characterized via immunofluorescence staining, and the expression of cardiac-specific genes was measured via RT-PCR. The functional assessment of SVF-derived cardiomyocyte-like cells (SVF-CMs) was performed by detecting cellular calcium transient activities and pharmacological responses. Results showed that most SVF-CMs exhibited elongated myotubule shapes and expressed cardiac troponin I strongly. SVF-CMs expressed cardiac-specific RNA (including transcription factors GATA binding protein 4) and myocyte enhancer factor 2c, as well as the structural proteins, namely, sarcomere actinin alpha 2, cardiac troponin I type 3, cardiac troponin T type 2, and cardiac gap junction protein alpha 1. Their beating mode, calcium activities, and pharmacological responses were similar to those of native CMs. Spontaneously beating SVF-CMs can be derived from adipose tissue-derived SVFs, and enzyme-crosslinked gelatin hydrogel promoted the cardiac differentiation of SVF cells.

Heart failure often develops after acute myocardial infarction because the injured myocardial tissue fails to recover or regenerate. Many efforts have been given to develop treatments for the repair of damaged heart and restoration of its function^1^. Therapeutic options include drug treatment, surgery, cardiac organ transplantation, and cell therapy. Stem cell therapy is progressing quickly as a promising treatment option in tissue engineering and regenerative medicine. However, a number of unresolved questions are related to stem cell handling and preparation, repair ability of the failing heart, and mode of cell delivery^2^. One of the fundamental questions is which cell type should be transplanted to obtain high efficiency and safety.

To date, the majority of clinical trials of cell therapy for heart failure mainly apply total bone marrow-derived mononuclear cells^3^. Nevertheless, these bone marrow-derived cells have limited ability to differentiate into cardiomyocytes (CMs) even after they are transplanted into the recipient myocardium. Hence, the most suitable stem cell therapy for heart failure is the application of cardiac-committed cells induced *in vitro*. Many examples of cell therapies using cardiac-committed cells show more considerable promising therapeutic effects compared with those cells that are not committed to a cardiac lineage, such as bone marrow mononuclear cells, mesenchymal stem cells, or skeletal myoblasts^4^,^5^,^6^,^7^. The superiority of cardiac-committed cells could be evidenced on the basis of various endpoints, such as good engraftment, reduced extent of infarction and fibrosis, increased angiogenesis and vasculogenesis, improved cardiac function, positive remodeling, and mitigation of ventricular arrhythmias through their electromechanical coupling with the recipient myocardium^2^.

Synthetically, beating cardiac-committed cells may play an important role in cardiac remodeling when transplanted in an ischemic heart because of their upregulated cardioprotection protein expression profile and the upregulation of antioxidant and cardiac remolding molecules^8^. To the best of our knowledge, the majority of stem cell-derived CMs with spontaneous beating capacity originated from embryonic stem cells (ESCs)^8^,^9^,^10^,^11^, induced pluripotent stem cells (iPSCs)^11^,^12^, cardiac progenitor cells (CPCs)^13^,^14^,^15^, and stromal vascular fraction (SVF)^16^,^17^,^18^. However, the sources of ESCs, iPSCs, and CPCs are confronted with controversy or have poor accessibility. For example, ESCs have ethical and legal issues; iPSCs are concerned with the safety of cell reprogramming and genetic engineering; and technical difficulties in cell collection are connected with CPCs.

SVF cells represent a heterogeneous cell population contained within adipose tissue that is commonly isolated using enzymes, such as collagenase. A mixed population that includes adipose-derived mesenchymal/stromal stem cells (ADSCs), endothelial precursor cells, T regulatory cells, macrophages, smooth muscle cells, pericytes, and preadipocytes was found with the removal of mature adipocytes, connective tissue, and blood cells from lipoaspirate, where SVF originated^2^. The difference between SVF cells and ADSCs is that SVF cells are the unexpanded cells while ADSCs are the expanded cells by cultivation^19^. Using SVF cells rather than ADSCs may demonstrate more significant benefits and healing potential because of the inclusion of heterogeneous cell types and autocrine growth factors^2^. SVF can differentiate into adipocytes, chondrocytes, osteoblasts, myocytes, cardiomyocytes, and hepatocytes, as well as neuronal, epithelial, and endothelial cells^20^,^21^.

The first protocol of SVF induction toward spontaneously beating CMs was described by Planat–Benard’s group^18^. The cell induction medium consists of semisolid methylcellulose medium, insulin, transferrin, and several expensive hematopoietic cytokines (i.e., interleukin (IL)-3, IL-6, and SCF)^18^. In the current study, we tried to simplify the induction procedure by substituting the semisolid culture with enzyme-crosslinked gelatin (gelatin/microbial transglutaminase (mTG)) hydrogel and using non-additive cytokines. Enzyme crosslink is a new method for biomaterial crosslink; mTG, which is derived from the streptomycete, has a high specific activity over a wide range of temperature, and pH and is Ca^2+^-independent^22^,^23^. mTG has been extensively utilized in the food industry^24^ and in tissue engineering for crosslinking cell scaffold, such as collagen and gelatin^25^,^26^.

In the current study, rat SVF cells were isolated and plated on gelatin/mTG hydrogels and subsequently induced with differentiated medium. Cell morphological features and spontaneous beating from differentiated cells were observed and recorded using biological microscopy. Cardiac phenotypes of SVF-derived cardiomyocyte-like cells (SVF-CMs) were characterized via immunofluorescent staining. In addition, the expression of cardiac-specific genes was measured by using real-time reverse transcription polymerase chain reaction (RT-PCR). The cardiac functional assessment for the differentiated cells was performed by detecting cellular calcium transient activities and pharmacological experiments.

## Results

### Morphological observation of cell growth and contractile activity

**Figure 1:**
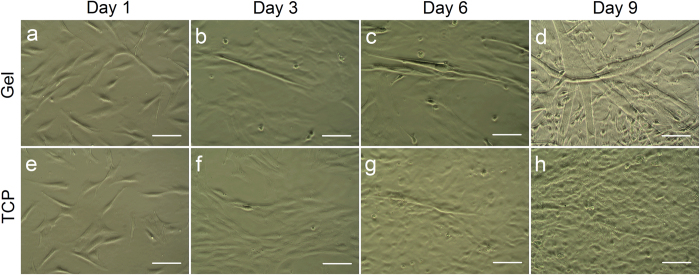
Morphological analysis of stromal vascular fraction (SVF) cells cultured on (**a**–**d**) gelatin/microbial transglutaminase (mTG) hydrogels and on (**e**–**h**) tissue culture plates (TCPs) in different time points over a two-week differentiated culture. Scale bar = 100 μm.

To investigate whether adipose tissue contains cells capable of differentiating into CMs, SVF cells were isolated from rat adipose tissue and cultured on gelatin/mTG hydrogels. After 24 h of culture, non-adherent cells, such as blood cells, lymphocytes, and adipocytes, were removed. The shape of the adherent cells was initially round and became polygonal or spindle-shaped thereafter (Fig. 1a). At day 3 after plating, myotube-like structures first appeared in the culture with the presence of hydrogel (Fig. 1b). These elongated cells grew in size and in number and were gradually surrounded with some round cells. In addition, some irregular tube-like structures appeared in the control group at day 6 (Fig. 1g), but later lost their tube-like shape with cell proliferation and confluence (Fig. 1h). At day 9 of culture, abundant myotubes with spreading branches and sharing tight junctions were observed in the culture with hydrogel. Some small and round cells also maintained growth in the culture (Fig. 1d). Altogether, these cell morphologies were similar to those of SVF cells reported in literature^16^,^17^,^18^.

The cell contractile activity started early at day 6 in some elongated cells and round cells cultured on gelatin hydrogels. In about 2 weeks, many contractile cell clusters could be observed under microscope. However, no contractile activities were found in the control groups. The developing process of cell beating in all observed SVF-CMs followed the same trend, in which spontaneous and independent beating typically begun with individual cells. Afterward, concurrent beating occurred after cell fusion and formed multinuclear myotube structures (Supplementary Information, Movie S1,2,3). To make a comparison, the continuous beating activities of native CMs was shown in a supplementary video (Supplementary Information, Movie S4).

### Immunofluorescent evidence of cell development into cardiac phenotypes

**Figure 2:**
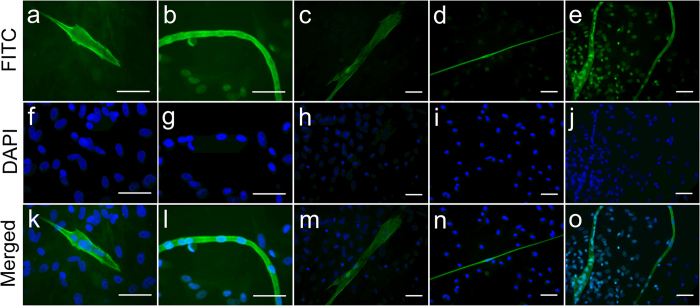
Immunofluorescence staining of SVF-derived cardiomyocyte-like cells (SVF-CMs). SVF cells were cultured on gelatin/mTG hydrogels for two weeks, and cardiac differentiation was detected via immunofluorescence staining of cardiac troponin I (cTnI). (**a**–**e**) The cTnI-positive cells were labeled in green through fluorescein isothiocyanate (FITC), a variety of cell shapes were found. (**f**–**j**) Cell nuclei were labeled in blue with 4,6-diamidino-2-phenylindole (DAPI). (**k**–**e**) Merged images of FITC and DAPI staining. Scale bar = 100 μm.

**Figure 3:**
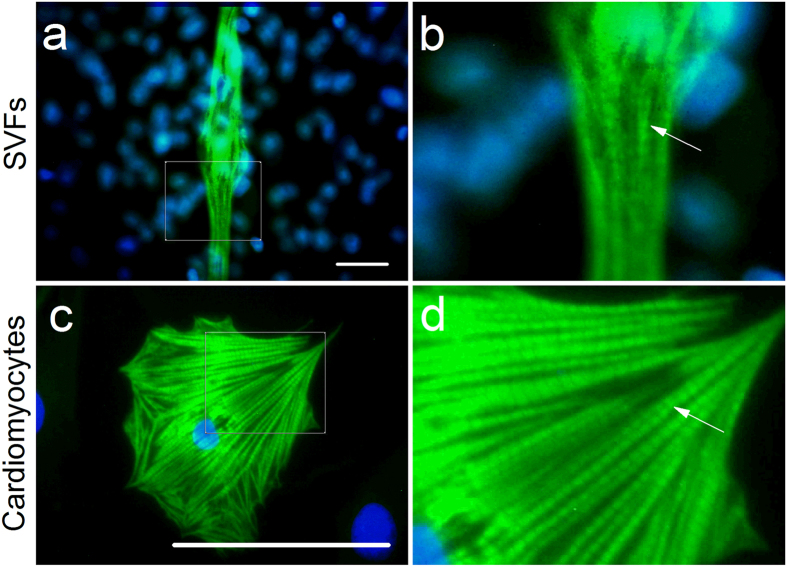
Sarcomeric structures were observed in SVF-CMs and native CMs. (**a**) Organized sarcomeric structure with intercalated disks was found in SVF-derived CMs. (**b**) An enlarged diagram of the white box region in panel “a”. (**c**) Organized sarcomeric structure with intercalated disks was observed in native CMs. (**d**) An enlarged diagram of the white box region in panel “c”. The cTnI-positive cells were labeled in green through FITC,staining and nuclei were labeled in blue with DAPI. White arrows indicated the intercalated disks. Scale bar = 100 μm.

To further define the phenotype of differentiated SVF cells, immunofluorescence staining of cardiac-specific cTnI was performed on the gelatin/mTG hydrogels with elongated clusters of cells and in control groups. cTnI-positive cells were labeled in green via FITC, and nuclei were labeled in blue with DAPI. Typical cell shapes, including leaf-like, string bean-like, fine thread-like, and spindle-like shapes with a multinuclear structure, were found under an immunofluorescence microscope (Fig. 2). cTnI expression was not only found in the nuclear-fused cells. but also in the nuclei around the SVFs, see Fig. 2b,e, Organized sarcomeric structure with intercalated disks could be observed in some mature SVF-CMs (Fig. 3a,b). To make an intuitive comparison, sarcomeric structure of native CMs in positive control group was shown in Fig. 3c,d. Of particular note is the cTnI-antibody utilized in this work had a very high cardiac specificity, because non-CMs were almost impossible to be stained. In Fig. 3c, a non-CM appears in the lower right corner, however, only the nucleus could be seen, its cytoplasm is barely visible. In addition, very few positive cTnI stainings in negative control groups could be observed. These observations indicated that SVF cells cultured on hydrogels had the potential to differentiate into CMs. To estimate the cardiac differentiation efficiency among the SVF cells, the percentages of cTnI^+^ cells versus total adherent cells were determined. The results indicated that 14.29 ± 3.29% cells cultured on gelatin/mTG hydrogels expressed cTnI. In control groups, the ratio of cTnI^+^ cells was only 1.36 ± 0.72%.

**Figure 4:**
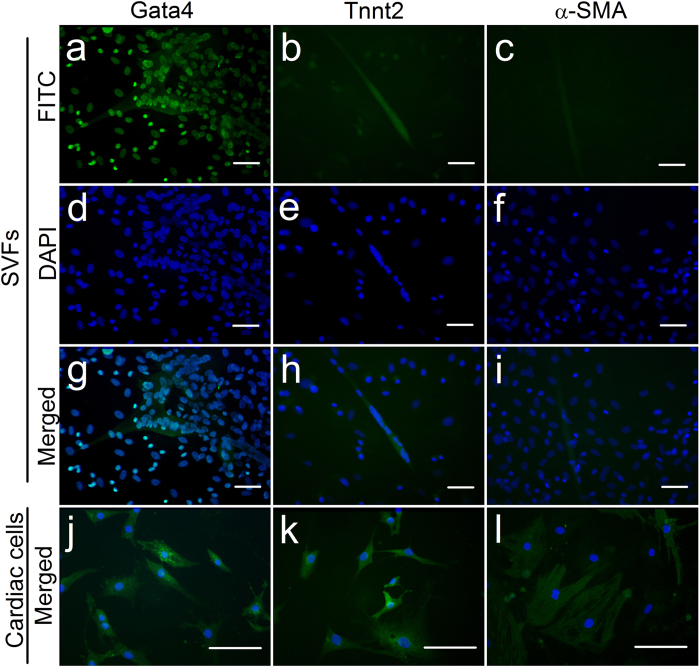
Immunofluorescence staining of SVF-CMs and native CMs. (**a**,**d**,**g**) Gata4 expression in SVFs. (**b**,**e**,**h**) Tnnt2 expression in SVFs. (**c**,**f**,**i**) α-SMA expression in SVFs. (**j**) GATA4 expression of native CMs. (**k**) Tnnt2 expression of native CMs. (**l**) α-SMA expression of native CMs. Antibody-positive cells were labeled in green through FITC staining, and nuclei were labeled in blue with DAPI. Scale bar = 100 μm.

Immunofluorescence staining of GATA binding protein 4 (Gata4), cardiac troponin T type 2 (Tnnt2), alpha smooth muscle actin (α-SMA) in SVFs and native CMs were also performed and the results were shown in Fig. 4. In SVFs, Gata4 expression was not only found in the nuclear-fused cells. but also in the nuclei around the SVFs, see Fig. 4a,d,g. As a contrast, Gata4 expression in native CMs is shown in Fig. 4j. The expression of Tnnt2 in SVFs could be detected, but the fluorescence intensity was much lower than that of cTnI, see Fig. 4b,e,h. Tnnt2 expression in native CMs is shown in Fig. 4k. The expression of α-SMA was very weak in SVFs, but still we could observe some faint antibody-staining in polynuclear cells. see Fig. 4c,f,i. Notably, native CMs were also stained by the α-SMA antibody, see Fig. 4l.

### RNA evidence of cell development into cardiac phenotypes

**Figure 5:**
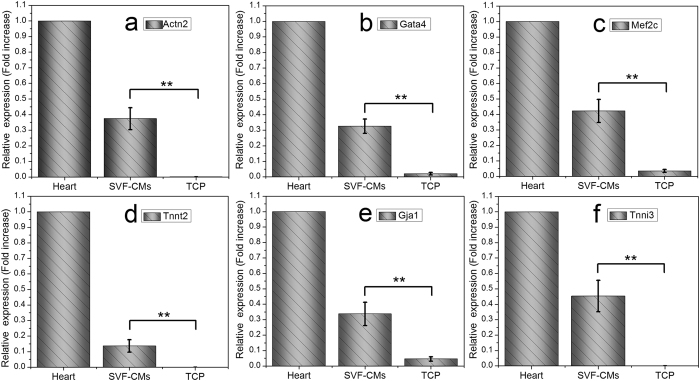
The expression levels of cardiac-specific genes. The expression level of cardiomyocyte markers (**a**. sarcomere actinin alpha 2, **b.** Gata 4, cardiomyocyte enhancer factor 2c, **d**. Tnnt2, **e**. cardiac gap junction protein alpha 1, **f**. cardiac troponin I type 3) of SVF cells cultured on gelatin/mTG hydrogels and on TCPs was evaluated by using reverse transcription polymerase chain reaction. RNAs from the rat heart were employed as positive control. All values were normalized to β-actin level. Gene expression was measured from six independent experiments (**p < 0.01).

To define the phenotypes of contracting cells at molecular level, the expression of cardiac-specific genes was measured via RT-PCR. Elongated clusters of SVF cells were collected from cell cultures, and total RNA was extracted, which was followed by PCR amplification. The expression of several cardiac-specific genes was assessed using RNAs from rat atrium and ventricle as positive control. Figure 5 shows that elongated clusters of SVF cells cultured on hydrogels expressed several cardiac-specific RNAs. Cardiac troponin I type 3 (cTnI type 3 or Tnni3) expression level was higher than that of other genes, but no more than a half of their counterpart expression level in the heart. The Actn2, Tnnt2, and Tnni3 expressions were not obvious in the control group, although weak expression of Gata4, Mef2c, and Gja1 was detected. These data suggested that elongated clusters of cells in SVF had preliminary differentiated into CMs, although the expression level of cardiac-specific markers had not yet competed with that of mature CMs. Moreover, the data indicated that the cell seeded on TCPs could not effectively differentiate into CMs with the current differentiation condition.

### Calcium fluorescence image analysis

**Figure 6:**
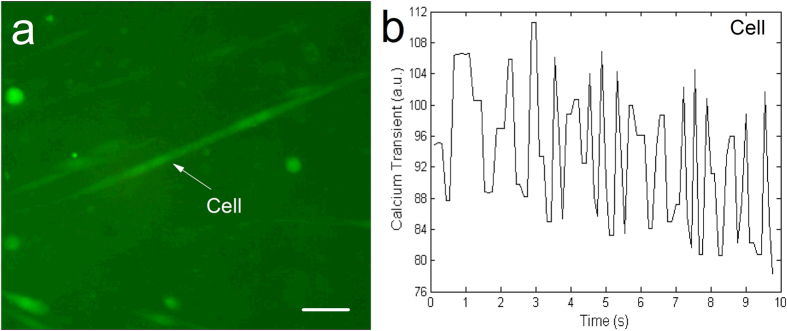
Spontaneous calcium transients analysis. (**a**) Ca^2+^ fluorescence image from SVF-CMs loaded with the Ca^2+^ indicator fluo-3/AM. (**b**) A calcium transient wave of the cell denoted by a white arrow in the panel “a” was produced through a customized MATLAB program. Scale bar = 50 μm.

Evidence of calcium activities in SVF cells cultured on hydrogels was characterized via Ca^2+^ fluorescence indicator fluo-3/AM. The studies were performed after two weeks of differentiation culture. Figure 6a shows both elongated and round cells labeled through the Ca^2+^ indicator. Spontaneous calcium transients were observed in many cells, including elongated and round cells. The calcium transient waves were independent of each other because these single cells had only started to beat, and their shared tight connections had not yet formed (Fig. 6b). Calcium transients were evoked spontaneously by Ca^2+^ release from intracellular Ca^2+^ stores without the synchronous signal of external pacing. Thus, the rhythm in these transient waves was fluctuant over time. Furthermore, calcium activities in cells were vulnerable to the strong fluorescence beams of microscope. Consequently, these calcium transient waves were easily decayed in the observation process. (Supplementary Information, Movie S5). Taken together, the results indicated that differentiated SVF cells had the function of calcium activity similar to that of native CMs.

### Pharmacological studies

**Figure 7:**
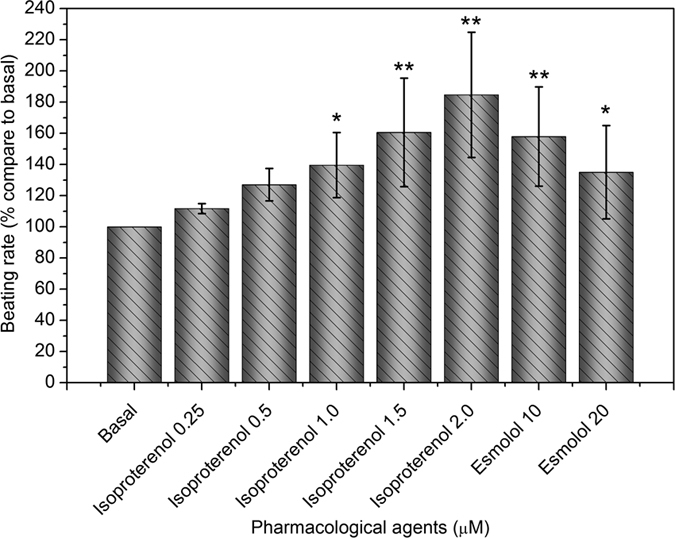
Chronotropic response of contractile SVF-CMs to adrenergic stimulation. SVF cells were cultured on gelatin/mTG hydrogels for two weeks. Subsequently, differentiated cells with regular contractile activity were observed. Cell beating rates were measured before and after treatment with different dosages of β-adrenergic agonist isoproterenol and antagonist esmolol. Results represent the mean of six individual experiments. (*p < 0.05, **p < 0.01).

To test the functionality of the differentiated SVF cells, we examined the chronotropic response of mature contracting SVF-CMs to the agents known to control the heart rate. Pharmacological studies were performed on beating cells with elongated morphology. The β-agonist isoproterenol (0.25–2 μM) induced a dose-dependent increase of the spontaneous contraction rate (Fig. 7). Esmolol (10–20 μM), a nonselective β-adrenergic antagonist, reversed the isoproterenol-induced acceleration (Fig. 7). These chronotropic responses suggested that the contracting SVF-CMs could respond to the adrenergic agents.

## Discussion

In the current study, rat SVFs cultured on gelatin/mTG hydrogels expressed CM-specific markers and demonstrated spontaneous beating behavior, cellular calcium transient activity, and pharmacological response similar to native CMs. This finding is valuable for the research in cardiac tissue engineering and regenerative medicine. A large amount of effort has been given on stem cell therapies of heart failure for many years. Although evolutional techniques and strategies are obtained annually, a primary hindrance is how to differentiate stem cells into cardiac-committed cells with a beating function *in vitro* before cell transplantation.

Cardiac-committed cells display more considerable therapeutic effects compared with those cells that are not committed to a CM fate. Currently, several stem cell types, such as ESCs, iPSCs, and CPCs, are the major sources of cardiac-committed cells with spontaneous beating capacity. However, each of these cell types has drawbacks in clinical applications. SVF is a promising cell source that has been utilized for obtaining spontaneously beating CMs *in vitro* in many studies^16^,^17^,^18^. Nevertheless, the cardiac induction conditions used in these studies consisted of semisolid methylcellulose medium, insulin, transferrin, and some hematopoietic cytokines, which are complex and expensive. Semisolid methylcellulose medium induces the formation of embryoid bodies from ESCs^27^, promotes the multilineage differentiation from murine adult pancreatic progenitor cells^28^, and enhances the contractile clone development of SVF-derived CMs^18^. Nonetheless, Planat-benard *et al*.^18^ reported that there was no definitive evidence that the semisolid medium was a key factor in the cardiac differentiation of SVF because SVF cells cultured in liquid medium could also be differentiated into beating cells despite that the occurrence of such event was rare.

In our study, the semisolid methylcellulose medium was substituted with gelatin/mTG hydrogel. Many types of hydrogel materials, including collagen hydrogel and gelatin hydrogel, promote cardiac differentiation *in vitro*^29^,^30^. Moreover, gelatin hydrogel can enhance the engraftment of transplanted CMs and angiogenesis to ameliorate cardiac function after myocardial infarction^31^. Gelatin/mTG hydrogels have a distinct advantage over synthetic hydrogel systems because of their superior biocompatibility and low costs. Transglutaminase is a natural protein present in several forms in mammalian tissue, blood, ECM, and on cell surfaces. This protein has a transamidation activity and catalyzes the formation of *N*-ε-(γ-glutamyl)lysine protein crosslink. mTG also has transamidation ability similar to that of the mammalian versions^23^. In our previous experiments, the gelatin hydrogel crosslinked via mTG was not toxic and can support the attachment and proliferation of ADSCs^32^. In this study, the shape of adherent SVF cells on gelatin/mTG hydrogel was initially round and became polygonal or spindle-shaped thereafter. Cell fusion and myotube-like multinuclear structure first appeared at day 3 after plating and gradually grew into contractile clusters with spreading branches and sharing tight junctions. The growth pattern of the cultured cells in this study is similar to that reported in the literature^16^,^17^,^18^,^33^,^34^. Furthermore, these elongated cells and many small and round cells accompanied the growth and development of these contractile clusters. Many elongated cells and some round cells exhibited beating behaviors and calcium transient activities.

Regarding the components of cardiac induction medium, we did not use chemical inducer 5-azacytidine as described for CM differentiation of many types of stem cells^35^,^36^ or any additive cytokine, growth factor, or chemokine. With regard to the role of several cytokines added in the semisolid methylcellulose medium for SVF cardiac differentiation, Planat–Benard *et al*. considered that these cytokines play a role in accelerating the SVF cardiac differentiation, but they are not the necessary additives. They observed that SVF cells could still grow into contractile clusters even without cytokine addition^18^. We speculate that SVF cardiac differentiation mainly relies on cell paracrine mechanism because SVF is a heterogeneous collection of cells releasing many kinds of factors, such as vascular endothelial growth factor, insulin-like growth factor-1, hepatocyte growth factor, transforming growth factor-b, basic fibroblast growth factor, IL-6, and IL-10^20^,^37^. These secreted factors promoted the minority populations of stem cells (stem and progenitor cells: <0.1%; stromal cells: 15%–30% in SVF^38^) to grow and differentiate toward CM fate. Ascorbic acid was the only additive we used in induction medium. Such acid can stimulate the ECM secretion of collagen and glycosaminoglycan, enhance the proliferation of MSCs^39^, and promote the cardiac differentiation of ESCs^40^.

In our experiments, SVF-CMs strongly expressed the cardiac-specific protein cTnI. Troponin is a complex of three regulatory proteins (cTnC, cTnI, and cTnT) that is integral to muscle contraction in skeletal muscle and cardiac muscle. Additionally, troponin facilitates the interactions between actin and myosin via binding to Ca^2+^, and cTnI is exclusively expressed in cardiac muscle. Over two weeks of culture, some mature SVF-CMs showed the sarcomeric-organized myofibrils, which were the specific characteristic of native CMs. Our findings are consistent with the features of SVF-derived CMs reported in the literature^16^,^17^,^18^. The percentage of cTnI-positive SVF-CMs from the SVF cultured on gelatin/mTG hydrogels was 14.29 ± 3.29%, and this ratio is comparable to that of Leobon’s study^17^, they reported that the percentage of cTnT-positive SVF-CMs obtained from the culture using semisolid medium was around 10%. However, after isolating the adherent elongated SVF cells and re-plating -on culture dishes supplemented with BHK21 medium for two weeks, they found that cTnT-positive cells in SVF-CM expansion could reach about 60%^17^. This result shows that SVFs possess cardiogenic potential similar to ESCs and iPSCs. Laflamme *et al*.^41^ found that the cardiomyocyte induction efficiency of ESCs from traditional embryoid body-based culture was usually less than 1%. By changing the culture method with special monolayer culture supplemented with activin A and bone morphogenetic protein 4, the cardiomyocyte induction efficiency was greater than 30%. The cardiomyocyte induction efficiency of human iPSCs was 30–70% in Uosaki *et al*.’s study^42^ and around 76.1% in Masumoto *et al*.’s study^43^.

The SVF-CMs in the current study also expressed several cardiac-specific proteins, including Gata4 and Tnnt2, and expressed cardiac genes. The mRNA expression levels of Actn2, Gata4, Mef2c, Gja1, Tnni3, and Tnnt2 were statistically increased when SVF cells were cultured on gelatin/mTG hydrogels. Together with the data of cellular calcium transient activities and pharmacological experiments, our finding showed that contractile cells had similar phenotypes and functions to those of native CMs, and enzyme-crosslinked gelatin hydrogels can promote the cardiac differentiation of SVF. Nevertheless, our study still has some limitations. First, although the elongated cell morphology of SVFs reported in the literatures had been confirmed as differentiated CMs^16^,^17^,^18^,^33^,^34^, we still feel that the polynuclear SVF-CMs resemble to smooth muscle cells in shape. Second, because some faint α-SMA antibody-staining existed in the polynuclear SVF cells, therefore, we could not excluded the possibility of differentiated smooth muscle cells from SVFs. Of course, native CMs were also stained by the α-SMA antibody, hence, the specificity of α-SMA needs further experimental screening and verification. Moreover, we speculate that there may be some common pathways for the differentiation of smooth muscle cells and the differentiation of CMs from SVFs. In this regard, further research is needed. Meanwhile, skeletal muscle phenotype in SVF-CMs likewise need a inspection to confirm cell differentiated direction.

In conclusion, we find a new approach to induce SVFs to the destination of CMs by using gelatin/mTG hydrogel culture. The experiment results of immunofluorescence staining of cardiac specific proteins and the mRNA expression levels of cardiac markers, plus the results of cellular calcium transient activities and pharmacological experiments, suggest beating CM-like cells can be derived from SVFs. However, the accurate mechanism of cell differentiation in SVF is not yet known, and the molecular and functional features in differentiating SVF-CMs need further studies in the future.

## Methods

### Preparation of gelatin hydrogels

Gelatin hydrogels were prepared as follows: 4% gelatin (type A, 300 Bloom; Sigma, MO, USA) solution was prepared via dissolving gelatin in phosphate-buffered saline (PBS) at 50 °C and immediately sterilizing it through 0.22 μm filters. Microbe transglutaminase (Bomei, China; enzyme activity, >100 U per gram) was prepared through dissolving mTG in PBS to obtain 10% (wt) solution and subsequently sterilizing it through 0.22 μm filters. Gelatin/mTG hydrogels were prepared via adding mTG into the gelatin solution at the proportion of 10 U/g·pro (enzymatic activity unit per gram of protein). Afterward, aliquots 2 ml of mixing solution were pipetted into the six-well tissue culture plates (TCPs) and incubated at 37 °C for 2 h. After gelation, these gelatin hydrogel-covered dishes were ready for cell culture.

### SVF isolation and cardiac differentiation

Animal study was approved by the Institutional Animal Care and Use Committee (IACUC) of Sichuan University, all experiments were performed in accordance with the guidelines of IACUC of Sichuan University. The inguinal adipose tissues were obtained from Sprague–Dawley rats (aged 4–6 weeks, either male or female). After the removal of blood vessels, these tissues were washed extensively with sterile PBS to remove contaminating debris and red blood cells. The tissue was minced mechanically and digested with 0.1% collagenase type I (Sigma, MO, USA) in high-glucose DMEM. Digestion was carried out under continuous agitation for 45 min at 37 °C, and the digested tissue was filtered and centrifuged at 283 g for 7 min. Cellular precipitation was harvested and resuspended with culture medium (high-glucose DMEM (Hyclone, UT, USA), which is supplemented with 15% fetal bovine serum (FBS, Gibco, NY, USA), 100 U/ml penicillin, and 100 μg/ml streptomycin (Hyclone, UT, USA)). For each digestion, 6 ml of SVF cell suspension was prepared. Subsequently, aliquots of 1 ml cell suspension were added into aforementioned hydrogel-covered TCPs and nonhydrogel TCPs as negative controls. After cell seeding, each dish was supplemented with the culture medium and incubated at 37 °C with 5% CO_2_.

After 24 h of culture, non-adherent cells were removed, and adherent cells were rinsed thrice with PBS. Afterward, cell differentiated medium was added into the dishes. The differentiated medium consisted of high-glucose DMEM, 10% FBS, 100 U/ml penicillin, 100 μg/ml streptomycin, and 50 μg/ml L-ascorbic acid. The cell culture was continued, and the differentiated medium was changed twice a week over a two-week experiment period, during which no trypsinization or cell passage was performed despite that complete cell confluence was obtained. The cells were observed daily under an inverted phase-contrast microscope (CKX41, Olympus, JAPAN). Furthermore, cell contractile activities and morphological developments were followed. At the end of cell cultivation, harvested cell samples were tested with the methods described in the following sections to evaluate the cardiac differentiation of SVF cells.

Native CMs were isolated using a protocol described in literature^44^. After 7–10 days culture on gelatin-coated coverslips, the samples of CMs were used as positive control groups in immunofluorescence staining. Observation and tracking of beating CMs were performed under an inverted microscope with an video recording system. Cell differentiation evaluation via immunofluorescence staining. The cardiac differentiation of SVF was characterized via immunofluorescence staining for cardiac-specific protein: cTnI, Tnnt2, Gata4, andα-SMA. Briefly, after two weeks of culture, SVF cells cultured on gelatin/mTG hydrogels or on TCPs were fixed with 4% paraformaldehyde for 30 min at room temperature and subsequently rinsed thrice with PBS. The fixed cells were blocked for 30 min in blocking solution (PBS supplemented with 2% normal goat serum, 1% bovine serum albumin, 0.1% Triton X-100) and incubated overnight at 4 °C with the primary antibodies of rabbit anti-cTnI (sc-15368, Santa Cruz, TX, USA), rabbit anti-Gata4 (sc-9053, Santa Cruz), goat anti-Tnnt2 (sc-8121, Santa Cruz), mouse anti-α-SMA (BM0002, Boster, China). After washing, the samples were incubated for 1 h with the secondary antibodies of fluorescein isothiocyanate (FITC) conjugated goat anti-rabbit IgG or donkey anti-goat IgG or rabbit anti-mouse IgG (Bethyl, TX, USA). Additional 1 μg/ml 4,6-diamidino-2-phenylindole (DAPI, Sigma, MO, USA) was added to stain nuclei. Samples were mounted over glass slide using antifade mounting medium (Beyotime, China). Native CMs mentioned above were also stained and used as a positive control group, SVFs culture on TCP were used as a negtive control group. Images were captured using a fluorescence microscope (BX60, Olympus, JAPAN) with a video camera (MD50, Mingmei, China). Ten high-magnification fields of each sample were chosen randomly. To estimate the efficiency of cardiac differentiation among the SVF cells, the percentages of cTnI-positive cell nuclei versus total cell nuclei were determined. The cells with a polynuclear structure are counted according to the number of their nuclei.

### Cell differentiation evaluation via RT-PCR

SVF cells were cultured on gelatin/mTG hydrogels or on TCPs in differentiation medium for two weeks. Subsequently, the expression of cardiac-specific genes, such as cardiac transcription factors Gata4, cardiac gap junction protein alpha 1 (Gja1), sarcomere actinin alpha 2 (Actn2), cardiac troponin I type 3 (Tnni3), Tnnt2, and myocyte enhancer factor 2c (Mef2c), was measured by using RT-PCR. For the sample preparation of SVF-CMs, elongated cells and contracting cell clusters cultured on hydrogels were dissected using a scalpel or a micropipette under an inverted phase-contrast microscope. For the sample preparation of SVF cultured on TCPs, cells were directly collected from TCPs using a cell scraper (Corning, NY, USA) without selection. Total RNA of the collected cells was extracted using TriZol reagent (Life Technologies, CA, USA), and RNAs from rat atrium and ventricle were adopted as positive control. Complementary DNA was synthesized from 1 mg of total RNA through employing RevertAid First Strand cDNA Synthesis Kit (K1622, Thermo Scientific, Lithuania). Complementary DNA samples were subjected to PCR amplification using Luminaris Color HiGreen qPCR Master Mix (K0391, Thermo Scientific, Lithuania). The primer sequences of each gene are listed in Table 1. Control reactions consisted of the above-mentioned PCR amplification were mixed with primers but without cDNA template. PCR was performed in six independent experiments using a RT-PCR detection system (iCycler IQ5, Bio-Rad, CA, USA). Cycles were programmed as follows: 95 °C for 10 min, 40 cycles of 15 s denaturation at 95 °C, 30 s at annealing temperature of 57 °C, 30 s extension at 72 °C, and a final extension at 72 °C for 10 min. The 2^−ΔΔCt^ method was used to evaluate the relative RNA expression levels for each target gene^45^. Expression of the housekeeping gene β-actin was employed for internal normalization. The product size was confirmed by running 10 μl of sample on 2% agarose gel electrophoresis.

### Image analysis of calcium transient

After two weeks of cardiac differentiation, SVF-CMs were prelabeled by incubation for 20 min at 37 °C in the modified Tyrode’s solution containing (in mmol/l): 136 NaCl, 5.4 KCl, 0.33 NaH_2_PO_4_, 1.0 MgCl_2_, 10 HEPES, 10 glucose, and 1.8 CaCl_2_ (pH 7.4), together with the Ca^2+^ indicator fluo-3/AM (10 μmol/l, AAT Bioquest, CA, USA). Incubation was followed by two 20 min washes in dye-free Tyrode’s solution. Fluorescence images were obtained using an inverted fluorescence microscope (XDS30, Sunny, China) operating in a video record mode (25 frames/s). Calcium transient wave was generated through a customized MATLAB (Mathworks, MA, USA) program. Briefly, a spontaneously beating cell was selected from a picture of the video frames, and the average brightness within the cell boundary was calculated frame-by-frame by the program. The temporal variations of brightness in the selected area corresponded to the calcium transients of the selected cell. Finally, the rhythmic wave of the cell was plotted precisely according to the time interval of video frames.

### Pharmacological Studies

For pharmacological treatments of SVF-CMs, elongated cells cultured on gelatin/mTG hydrogels with a regular contractile activity were selected for observation under an inverted phase-contrast microscope with a video capture camera. The basal beating rate of the observed cells was counted before and after the replacement of culture medium with the fresh DMEM-HG medium containing 10% FBS. Chronotropic responses were assessed by recording the variation of beating rate in the presence of the appropriate drugs. Dose response experiments were performed via adding agonist of 0.25–2 μmol/l isoproterenol (Qilu Pharma, China) or antagonist of 10–20 μmol/l esmolol (Qilu Pharma, China). Antagonist was added after the maximal dose of agonist. A total of six individual experiments were performed. For statistical analysis, the basal beating rate of each measurement was normalized to 100%, and the percentages of changed beating rate were calculated.

### Statistical analysis

Data are presented as mean ± SD. Statistical analyses were performed with SPSS 13.0 software. Statistical significance was evaluated using one-way ANOVA with least-significant difference test. The differences with values of *p < 0.05* were considered statistically significant.

## Additional Information

**How to cite this article**: Yang, G. *et al*. Obtaining spontaneously beating cardiomyocyte-like cells from adipose-derived stromal vascular fractions cultured on enzyme-crosslinked gelatin hydrogels. *Sci. Rep.* **7**, 41781; doi: 10.1038/srep41781 (2017).

**Publisher's note:** Springer Nature remains neutral with regard to jurisdictional claims in published maps and institutional affiliations.

## Supplementary Material

Supplementary Video 1

Supplementary Video 2

Supplementary Video 3

Supplementary Video 4

Supplementary Video 5

Supplementary Videos

## Figures and Tables

**Table 1:** Sequences of polymerase chain reaction (PCR) primers.

Gene	Primer sequence (5′–3′)	Accession No.	Product (bp)
Tnni3	F: TGACCTGCGTGGCAAGTT	NM_017144.2	153
	R: TCCTTCTCAATGTCCTCCTTCT		
Actn2	F: AGAGAATGAGAGGCTGATGGA	NM_001170325.1	165
	R: GGCTTGTGCTTACGACGATA		
Mef2c	F: CGGACTGATGAAGAAGGCTTAT	XM_003749164.1	254
	R: GGCTGTGACCTACTGAATCG		
Cx43	F: TGTGATGAGGAAGGAAGAGAAG	NM_012567.2	192
	R: TTGAAGAGGATGCTGATGATGT		
Gata4	F: GCAGCAGCAGTGAAGAGAT	NM_144730.1	177
	R: GAGTGACAGGAGATGGATAGC		
Tnnt2	F: AGGAGGAAGGCTGAAGATGA	NM_012676.1	123
	R: CTCTCGCTCTGTCTGTCTCT		
β-Actin	F: GGACCTGACAGACTACCTCAT	NM_031144.3	217
	R: GAACCGCTCATTGCCGATA		
