# Supplementary material for: Obtaining spontaneously beating cardiomyocyte-like cells from adipose-derived stromal vascular fractions cultured on enzyme-crosslinked gelatin hydrogels
Source: Sci Rep. 2017 Feb 3;7:41781. doi: 10.1038/srep41781 (PMC5290532; doi:10.1038/srep41781)
Supplement: Supplementary Videos [file srep41781-s6.doc]

**Manuscript title:**

**Obtaining spontaneously beating cardiomyocyte-like cells from adipose-derived stromal vascular fractions cultured on enzyme-crosslinked gelatin hydrogels**

Gang Yang^1,*,+^, Zhenghua Xiao^2,+^, Xiaomei Ren^1^, Haiyan Long^3^, Kunlong Ma^4^, Hong Qian^2^, Yingqiang Guo^2,*^

^1^ Department of Medical Information and Engineering, School of Electrical Engineering and Information, Sichuan University, Chengdu 610065, China

^2^ Department of Cardiovascular Surgery, West China Hospital, Sichuan University, Chengdu 610041, China.

^3^ Center of Engineering-Training, Chengdu Aeronautic Polytechnic, Chengdu 610100, China

^4^ Department of Orthopaedics, Yongchuan Hospital, Chongqing Medical University, Chongqing 402160, China

^*^corresponding. yang_gang@scu.edu.cn (G.Y.), drguoyq@hotmail.com (Y.Q.G).

^+^these authors contributed equally to this work

**Supplementary Information**

**Movie S1. Video 1 of spontaneously beating SVF-CMs.** SVF cells were cultured on microbe transglutaminase-crosslinked gelatin (gelatin/mTG) hydrogels for 2 weeks, cell contractile activity was observed and recorded under an inverted phase-contrast microscope (CKX41, Olympus, JAPAN) with a video camera (MD50, Mingmei, China).

**Movie S2. Video 2 of spontaneously beating SVF-CMs.** SVF cells were cultured on gelatin/mTG hydrogels for 2 weeks, cell contractile activity was recorded.

**Movie S3. Video 3 of spontaneously beating SVF-CMs.** SVF cells were cultured on gelatin/mTG hydrogels for 2 weeks, cell contractile activity was recorded.

**Movie S4. Video 4 of spontaneously beating native CMs.** Native CMs were cultured on TCP for 10 days, cell contractile activity was recorded.

**Movie S5. Video record of calcium transient waves of spontaneously beating SVF-CMs.** SVF-CMs were prelabeled with Ca^2+^ indicator fluo-3/AM, fluorescence images were obtained using an inverted fluorescence microscope (XDS30, Sunny, China) operating in a video record mode (25 frames/s).
